# Association between cannabis use and treatment outcomes in patients receiving methadone maintenance treatment: a systematic review protocol

**DOI:** 10.1186/s13643-016-0317-2

**Published:** 2016-08-16

**Authors:** Laura Zielinski, Meha Bhatt, Rebecca B. Eisen, Stefan Perera, Neera Bhatnagar, James MacKillop, Meir Steiner, Stephanie McDermid Vaz, Lehana Thabane, Zainab Samaan

**Affiliations:** 1 MiNDS Neuroscience Graduate Program, McMaster University, 1280 Main Street West, Hamilton, ON L8S 4L8 Canada; 2 Health Research Methodology Graduate Program, McMaster University, 1280 Main Street West, Hamilton, ON L8S 4L8 Canada; 3 Department of Clinical Epidemiology and Biostatistics, McMaster University, 1280 Main Street West, Hamilton, ON L8S 4L8 Canada; 4 Health Science Library, McMaster University, 1280 Main Street West, Hamilton, ON L8S 4L8 Canada; 5 Department of Psychiatry and Behavioural Neurosciences, McMaster University, 1280 Main Street West, Hamilton, ON L8S 4L8 Canada; 6 Peter Boris Centre for Addictions Research, St. Joseph’s Healthcare Hamilton, 100 West 5th Street, Hamilton, ON L8N 3K7 Canada; 7 Women’s Health Concerns Clinic, St. Joseph’s Healthcare Hamilton, 100 West 5th Street, Hamilton, ON L8N 3K7 Canada; 8 Department of Obstetrics and Gynecology, McMaster University, 1280 Main Street West, Hamilton, ON L8S 4L8 Canada; 9 Cleghorn Early Intervention Clinic, St. Joseph’s Healthcare Hamilton, 50 Charlton Avenue E., Hamilton, ON L8N 4A6 Canada; 10 Biostatistics Unit, Research Institute at St Joes, St. Joseph’s Healthcare Hamilton, 50 Charlton Avenue E., Hamilton, ON L8N 4A6 Canada; 11 Population Genomics Program, Chanchlani Research Centre, McMaster University, 1280 Main Street West, Hamilton, ON L8S 4L8 Canada; 12 Mood Disorders Program, St. Joseph’s Healthcare Hamilton, 100 West 5th St., Hamilton, ON L8N 3K7 Canada

**Keywords:** Methadone maintenance treatment, MMT, Cannabis, Marijuana, Systematic review, Protocol

## Abstract

**Background:**

With the non-medical use of prescription opioids increasingly becoming a method of abuse in Canada, the number of patients requiring methadone maintenance treatment (MMT) for opioid use disorder has increased dramatically. The rate of cannabis use in this population is disproportionately high (~50 %). Because its use is generally perceived as harmless, cannabis use is often not monitored during MMT. Current literature regarding the effects of cannabis use on MMT is conflicting, and the presence and nature of an association has not been clearly established. The primary objective of this review will be to conduct a systematic review of the literature and, if appropriate, a meta-analysis to determine whether there is an association between cannabis use and MMT outcomes. A secondary objective will be to perform subgroup analyses (by age, sex, method of cannabis measurement, and country) to determine whether cannabis use differentially influences MMT outcomes within these subgroups.

**Methods/design:**

The search will be conducted on the following electronic databases using a predefined search strategy: MEDLINE, EMBASE, PsycINFO, and Cumulative Index to Nursing and Allied Health Literature (CINAHL). Two authors (LZ and MB) will independently screen articles using predetermined inclusion/exclusion criteria and will extract data from included articles using a pilot-tested data extraction form. Disagreements at all stages of the screening process will be settled through discussion, and when consensus cannot be reached, a third author (ZS) will be consulted. An assessment of quality and risk of bias will be conducted on all included articles, and a sensitivity analysis will be used to compare results of studies with high and low risk of bias. We will perform random- and fixed-effects meta-analyses, if appropriate, with heterogeneity calculated using the *I*^2^ statistic and formal evaluation of publication bias.

**Discussion:**

Results of this systematic review will elucidate the association between cannabis use and methadone maintenance treatment outcomes. We will provide evidence that will be useful to clinicians regarding whether monitoring cannabis use during MMT is advantageous for optimizing MMT outcomes.

**Systematic review registration:**

PROSPERO CRD42015029372

**Electronic supplementary material:**

The online version of this article (doi:10.1186/s13643-016-0317-2) contains supplementary material, which is available to authorized users.

## Background

There are an estimated 33 million opioid users globally [1], which is markedly contributing to the global burden of disease [2]. Illicit opioid use is associated with significant personal health risks, such as accidental injury, dependency, infectious disease [3], and potential for fatal overdose, in addition to its effects on the social concerns of healthcare costs, criminal activity, and employment [2]. Although the prevalence of heroin use has remained constant in Canada, a dramatic rise in the use of prescription opioids has resulted in a surge in opioid detoxification admissions from 2000 to 2004 [4]. In both the USA and Canada, illicit use of prescription opioids has become a significant contributor to emergency room visits and mortality [5]. This changing landscape and steady increase in problematic opioid use in North America signals an urgent need for evidence-based treatment practices.

Canada has witnessed a fivefold increase in patients on methadone maintenance treatment (MMT) since the mid-1990s [6]. MMT is an opioid substitution therapy and is the most widely researched pharmacological treatment for opioid use disorder [7]. Methadone is a long-acting synthetic opioid intended to reduce cravings and withdrawal symptoms without producing the euphoric effects associated with illicit opioids [8]. Studies have shown this treatment to be effective in decreasing illicit opioid use, reducing criminal activity, and reducing mortality rates among patients [7]. Although an overall efficacious treatment for reducing illicit opioid use, MMT is limiting in that it has high attrition rates [9] because it often requires patients to be on the treatment for life [10].

Polydrug use is common among MMT patients [11, 12], with cannabis consistently being the most commonly used illicit drug in this population [13–15]. This may be due to the fact that cannabis is generally perceived as harmless [16]. While it may be the case that mortality directly resulting from cannabis use is unlikely [2], associations with other adverse health outcomes have been found. In particular, regular cannabis use increases the risk of motor vehicle accidents and respiratory problems and poses a risk for dependency [17]. Long-term use is also associated with lower school performance and decreased life satisfaction [16]. Furthermore, cannabis use is associated with adverse mental health outcomes. The strongest evidence comes from studies on psychotic disorders, with a systematic review showing a strong, positive relationship between incidence of psychosis and cannabis use, which increases as frequency of cannabis use increases [18]. Studies have also found associations with other psychiatric illnesses including mood disorders (unipolar and bipolar) [19–21] and anxiety (particularly panic disorder and social anxiety) [22, 23]; however, evidence for a directional association with these disorders is inconclusive. Nonetheless, it remains a possibility that cannabis use during the treatment of opioid addiction could influence its outcome.

Studies on the association between cannabis use and MMT outcomes have produced conflicting results, with some demonstrating beneficial effects on outcomes [14] and others showing an association with adverse treatment outcomes. For example, Wasserman et al. [24] found that cannabis use at baseline and throughout the study period was significantly associated with subsequent heroin use during treatment, whereas Scavone et al. [14] found that patients using cannabis during the study reported significantly less daily expenditure on acquiring opioids. Most studies, however, have failed to produce a statistically significant association between cannabis use and MMT retention or illicit opioid use [13, 25–28]. Epstein and Preston [26] found that cannabis use increased other outcomes such as jail time and family conflict, suggesting that its use during MMT may act indirectly via social and lifestyle risk factors. The relationship between cannabis use and MMT outcomes may also include complex interactions with health behaviors. For instance, depressive symptoms and illicit substance use during MMT is significantly associated with a lack of HIV medication adherence [20], which may in turn affect MMT outcomes and overall health status among patients.

It remains unclear whether there is a true association between cannabis use and MMT outcomes and to what degree this association may be mediated by other confounding variables. A systematic investigation and evaluation of the studies is necessary, as well as the identification of any gaps in the literature. We hypothesize that the use of cannabis in patients with opioid use disorder treated with methadone is associated with poor response to MMT as defined by illicit opioid use and length of treatment retention. Evidence indicates that treatment retention is a critical factor in MMT success, with research suggesting that those in treatment for less than 90 days resemble those receiving no treatment at all [29]. Indeed, MMT dropout is significantly associated with drug use relapse and other high-risk health behaviors [11] and is a useful indicator of treatment response. We will also consider secondary outcomes to evaluate risky health and social behaviors including criminal activity, jail time, polydrug use, injection drug use, needle sharing, and unprotected sex. Isolating each outcome and controlling for potential confounders will help to clarify the association between cannabis use and MMT outcomes.

### Objectives

The objective of this systematic review is to summarize the existing literature examining the effects of cannabis use on treatment outcomes during methadone maintenance treatment in patients with opioid use disorder by identifying and evaluating the current evidence.

Specifically, our aims are as follows:

1. Summarize primary research to examine the relationship between cannabis use and primary methadone maintenance treatment outcomes (treatment retention and illicit opioid use) and secondary outcomes (criminal activity, jail time, polydrug use, injection drug use, needle sharing, and unprotected sex).
2. Combine statistical outcomes of the primary studies in a meta-analysis, when appropriate.
3. Conduct subgroup analyses based on sex, method of cannabis measurement, and geographical region of study to explore potential confounders in the relationship.
4. Critically appraise the existing literature and identify areas requiring further research.

## Methods and design

### Search strategy

An experienced health sciences librarian (NB) will be consulted when creating and implementing the search strategy. The following databases will be searched from their inception to present: MEDLINE/PubMed, EMBASE, PsycINFO, and Cumulative Index to Nursing and Allied Health Literature (CINAHL). Relevant articles will be identified from the comprehensive search strategy using all relevant search terms related to methadone maintenance treatment and cannabis, and their medical subject heading (MeSH) equivalents in varying combinations (Table 1). A wide search will be conducted to include titles, abstracts, and keyword fields. Outcome variables will not be included in the search strategy so as not to impose unnecessary limitations on search results. The searches will all be limited to human studies. Gray literature will also be searched using ProQuest Dissertations and Theses Global database. Finally, we will conduct a thorough hand search of past reviews and reference lists of included studies to identify potentially relevant articles that the initial search strategy may not have captured.

**Table 1:** Search strategy

Database	Search strategy
MEDLINE (*n* = 420)	1. exp Opiate substitution therapy/ 2. Methadone/ 3. Methadone.mp. 4. MMT.mp. 5. Cannabis/ 6. Marijuana Abuse/ 7. Marijuana Smoking/ 8. Medical Marijuana/ 9. Cannabis.mp. or marijuana*.mp. 10. THC.mp. or hash*.mp. or ganja.mp. or hemp.mp. or bhang*.mp. 11. 1 or 2 or 3 or 4 12. 5 or 6 or 7 or 8 or 9 or 10 13. 11 an 12 14. Limit 13 to humans
EMBASE (*n* = 1761)	1. exp opiate substitution treatment/ 2. exp methadone treatment/ 3. exp methadone/ 4. Methadone.mp. 5. MMT.mp. 6. exp cannabis/ 7. exp “cannabis use”/ 8. exp cannabis addiction/ 9. exp cannabis smoking/ 10. exp medical cannabis/ 11. Cannabis.mp. or marijuana*.mp. 12. THC.mp. or hash*.mp. or ganja.mp. or hemp.mp. or bhang*.mp. 13. 1 or 2 or 3 or 4 or 5 14. 6 or 7 or 8 or 9 or 10 or 11 or 12 15. 13 and 14 16. Limit 15 to humans
PsycINFO (*n* = 194)	1. exp methadone maintenance/ 2. methadone.mp. 3. MMT.mp. 4. exp cannabis/ 5. exp marijuana usage/ 6. cannabis.mp. or marijuana*.mp. 7. THC.mp. or hash*.mp. or ganja.mp. or hemp.mp. or bhang*.mp. 8. 1 or 2 or 3 9. 4 or 5 or 6 or 7 10. 8 and 9 11. Limit 10 to humans
CINAHL (*n* = 50)	1. (MH “Methadone”) 2. “Methadone” 3. “MMT” 4. (MH “Cannabis”) 5. (MH “Medical Marijuana”) 6. “marijuana” or “cannabis” 7. “THC” or “hash*” or “ganja” or “hemp*” or “bhang*” 8. 1 or 2 or 3 9. 4 or 5 or 6 10. 7 and 8 (limiters – human)

### Inclusion/exclusion criteria

This review will include published observational studies or randomized controlled trials (RCTs) of the relationship between cannabis use and methadone maintenance treatment outcomes in any setting (hospital, outpatient, or community-based). Included studies will measure cannabis use at baseline for cross-sectional studies and during treatment for cohort studies and RCTs, which may be measured using objective measures (i.e., urine or hair analyses) or self-reports.

Studies will be excluded if they do not measure at least one of the primary or secondary outcomes of interest. If cannabis use is measured as an outcome rather than a predictor variable, it will be excluded. Many studies in this domain report frequency of cannabis use as part of the demographics of the sample, and as such, these will be excluded as we cannot make any conclusions regarding its direct association with MMT outcomes. Studies including patients on opioid substitution therapy (i.e., buprenorphine or buprenorphine/naloxone) other than methadone will be excluded. Furthermore, studies looking at patients on methadone for anything other than treatment of opioid use disorder (i.e., illicit methadone use or chronic pain treatment) will be excluded. No age restrictions will be applied, as opioid addiction affects people of all age groups. There will be no other demographic limitations or language restrictions.

### Outcome measures

Two primary outcomes variables will be measured which evaluate the success of methadone maintenance treatment. These include illicit opioid use which may be measured in any way (self-reports, urine toxicology, hair analysis), as well as treatment retention. Treatment retention may be measured as either proportion of individuals remaining in treatment at the end of study or average length of time in treatment. In addition to the MMT outcomes, secondary outcomes will be considered which reflect the patients’ social and personal functioning and other drug use behaviors. These include criminal activity, jail time, polydrug use, injection drug use, needle sharing, and unprotected sex.

### Data management

All articles retrieved during the initial search will be uploaded to Covidence, an online software system used to manage systematic reviews and promotes collaboration among authors. Training will be provided to all members using the Covidence software. The review team will define a set of inclusion and exclusion criteria, and pilot test the title/abstract screening with the first 100 articles. Upon completion of title and abstract screening, full-text articles will be uploaded to the Covidence system for purposes of the full-text review.

### Selection of studies

Two independent reviewers (LZ and MB) will complete the initial title and abstract screening to identify eligible articles using a predetermined criteria. Articles deemed eligible will be retrieved for a full-text review. Any disagreements during the screening process that cannot be settled through discussion will be resolved by a third party (ZS). Authors of the studies will be contacted if any clarification or additional data is needed during the full-text review to determine eligibility. For each phase of screening, a kappa statistic will be calculated to determine inter-rater agreements. A kappa value of 0.75 or greater reflects excellent agreement [30]. Studies determined to be ineligible will be excluded from the review. Reasons for ineligibility and exclusion will be reported using the preferred reporting items for systematic reviews and meta-analyses (PRISMA) [31] or meta-analysis of observational studies in epidemiology (MOOSE) [32] flow diagrams.

### Data extraction

The two reviewers (LZ and MB) will independently extract data from the included studies using a pilot-tested data extraction form (see Additional file 1). To maximize consistency, a calibration exercise will be performed using articles not included in the review with the two reviewers prior to starting the data extraction phase. The authors will extract the following information from each study: publication details (name, author, year, journal, and country), study design (type of study, participant information, inclusion criteria, and length of study), demographics (mean age, ethnicity, and sex), measurement of cannabis (self-report, urinalysis, or hair analysis), outcome measures, the main findings, and statistical results (effect measures, *p* values, confidence intervals, etc.). If multiple outcomes are reported, all of them will be recorded so we can combine the studies with similar outcome measurements. Authors will be contacted in the case of missing or incomplete data.

### Assessment of quality

Risk of bias in will be assessed by two independent raters (LZ and MB) using the Newcastle-Ottawa Scale (NOS) [33]. An adapted version of a modified NOS was developed by Bawor et al. to be used to assess risk of bias in observational studies [34]. This version includes seven questions evaluating bias in four domains of biases: selection bias, performance bias, detection bias, and information bias. Risk of bias is measured on a scale of 0 (high risk) to 3 (low risk). The adapted version has removed items regarding the comparability of groups and suitable follow-up for cohort and case-control studies, as these items are not relevant for our topic of interest. The Cochrane Collaboration tool will be used for randomized controlled trials which assess risk of bias using six domains: selection bias, performance bias, detection bias, attrition bias, reporting bias, and other biases [35].

Quality of the literature will be measured using the grading of recommendations, assessment, development, and evaluation (GRADE) framework, which scores articles based on five domains—risk of bias, publication bias, consistency, directness, and precision [36]. These findings will be summarized in a table, allowing for an assessment of the confidence of the estimates. A summary of the findings will be provided in a table to easily compare the quality of studies included in this review and allow for confidence of estimates.

### Statistical analyses

All included studies will first be reviewed in a qualitative summary, followed by a meta-analysis if possible. Studies will be combined in a meta-analysis based on similarities in study design and outcome measurements. Direct estimates will be pooled separately based on study design, as pooling data from observational studies and RCTs is cautioned against due to the inherent susceptibility of observational studies to selection biases [37]. A random-effects model for meta-analysis will be used to account for the expected heterogeneity in the literature, which assumes both within-study and between-study variability to provide a more conservative estimate compared to a fixed-effects model. These results will be presented in a forest plot. In the case of missing data, we will attempt to contact authors to obtain the relevant data. If the missing data cannot be obtained, we will employ an imputation method. We may also conduct a sensitivity analysis to assess the impact of missing data on the overall treatment effects. A sensitivity analysis will also be conducted to compare the overall results of studies with high or low risk of bias.

Heterogeneity will be calculated among pooled studies using the *I*^2^ statistic. It is advised not to impose cut-off values because the importance of heterogeneity depends on a multitude of factors. However, Cochrane suggests that a value <40 % may not represent a notable amount of heterogeneity [37]. Thus, possible sources of clinical heterogeneity will be examined given an *I*^2^ statistic > 40 %, and subgroup analyses will be performed. Possible sources of heterogeneity include age, sex, method of cannabis measurement, and country, and these will be investigated using subgroup analyses.

### Subgroup analyses

Subgroups are identified a priori so we can make stronger inferences about the effects of the subgroups [38]. Subgroup analyses will be conducted on the following variables: age, sex, method of cannabis measurement, and country. Drug addiction is a disorder that afflicts people of all ages, and thus no age restrictions will be placed on the articles in this review. However, there are differences in the biological and social mechanisms involved with youth and adults, so cannabis may differentially influence treatment outcomes in the two populations. Because a consistent age range is not used to define youth in MMT studies [28, 39, 40], MMT studies will be included in the subgroup analysis if the authors specify that they are investigating youths or adolescents.

Methadone maintenance treatment has been found to differentially affect men and women [34], and prevalence of cannabis use tends to be higher in males [41]. However, females display a stronger dose-dependent effect from cannabis compared to males, with significantly lower mental quality of life as dosage increases [42]. Furthermore, women demonstrate a faster trajectory towards the development of cannabis use disorder [43]. Thus, particularly among heavy cannabis users, we expect treatment outcomes in women to be more negatively impacted by cannabis use. Stratifying these populations using a subgroup analysis may reveal differences in the way cannabis use affects MMT outcomes for males and females.

We will also compare results of studies that use subjective or objective measures of drug use. Studies have shown that a large number of patients in treatment for addiction underreport drug use [44], whether intentionally or not, and thus objective measures of drug use, such as urine or hair analysis, may provide a more accurate estimate of cannabis use in the population. Therefore, we expect to find a stronger association between objective measures of cannabis and MMT outcomes compared to studies using subjective measures.

Finally, any potential differences found between studies from different regions of the world or different decades will be qualitatively commented on and compared to current literature on drug use patterns considering the varied pattern of drug use across the world [2]. Specifically, North America has the highest proportion of cannabis use and high rates of opioid use, largely due to the surge in non-medical use of prescription opioids [1]. Illicit drug use is considerably less in Europe, with lower rates of cannabis use compared to North America, as well as significantly less opioid use [1]. On the other hand, more than half of the world’s opioid-using population lives in Asia, although cannabis rates are below the global average [1]. These different patterns of illicit drug use around the world signify that different societal mechanisms are at play, which may impact treatment outcomes for drug addiction.

### Presenting and reporting of results

This systematic review will be reported in accordance with the PRISMA guidelines [31]. Additionally, we expect to include many observational studies, in which case these will be reported following the MOOSE guidelines [32]. A flow chart will be used to display the selection of articles with reasons for exclusion. Study characteristics and measured outcomes will be compiled into summary tables. An Egger’s plot will be included to examine potential publication bias in the selected studies. If a meta-analysis is possible, results will be presented in a forest plot. The current protocol follows the preferred reporting items for systematic review and meta-analysis protocols (PRISMA-P) statement (see Additional file 2 for checklist) [45].

## Discussion

Using evidence from this systematic review, we expect to make conclusions regarding the presence of an association between cannabis use and methadone maintenance treatment outcomes. Systematically reviewing the literature will contribute to our understanding of the mechanisms involved in treatment retention and drug relapse in patients with opioid use disorder. We will also be investigating cannabis use and its association with other outcomes related to overall social and physical well-being in MMT patients.

To our knowledge, this will be the first systematic review conducted on this topic. Given the current trend of cannabis being approved in many US states and the move towards a more liberal use in Canada, it is imperative that these policy decisions are evidence-based. The findings of this systematic review will also be of value to clinicians administering methadone maintenance treatment to patients with opioid use disorder, as it will provide evidence regarding whether monitoring cannabis use during MMT is necessary, and how it may predict a patient’s treatment outcomes.

## Additional files

Additional file 1: Data extraction form. (PDF 7 kb) Additional file 2: PRISMA-P Checklist. (PDF 279 kb)

## Abbreviations

MMT: Methadone maintenance treatment
MOOSE: Meta-analysis of observational studies in epidemiology
NOS: Newcastle-Ottawa Scale
PRISMA: Preferred reporting items for systematic reviews and meta-analyses
PRISMA-P: Preferred reporting items for systematic review and meta-analysis protocols
RCT: Randomized controlled trial
