# Supplementary material for: Association between cannabis use and treatment outcomes in patients receiving methadone maintenance treatment: a systematic review protocol
Source: Syst Rev. 2016 Aug 16;5:139. doi: 10.1186/s13643-016-0317-2 (PMC4988054; doi:10.1186/s13643-016-0317-2)
Supplement: Additional file 1: — Data extraction form. (PDF 7 kb) [file 13643_2016_317_MOESM1_ESM.pdf]

Data Extraction Form

Study ID: ______________ Reviewer Initials: ________

Publication Details

Author (last name, first initial): ________________________________ Year: ________________
Title: ____
Journal: _____________________________________ Country: ____________________________

Methods

Study design: ______________________ Study setting: ___________________________________
Length of study: _______________________
Description of sample:
____
Exposure: __________________________________ Intervention (if applicable): _______________

Demographics

Number of participants: Total: _____ Men: _____ Women: _____ Per group: _______________
Mean age (SD): Total: ___________ Men: _________ Women: __________
Per group: ____
Ethnicity:
____

Exposure measurements (cannabis). Circle one.

Self-report Urinalysis Hair analysis Other _______________________________
Comments:
____

Outcome measurements:

Illicit opioid use: ____
Treatment retention: ____
Criminal activity: ____
Jail time: ____
Polydrug use: ____
Injecting behaviours: ____
Comments:
____

Results

Statistical methods: ________________________________ Adjusted for: ____________________
Coefficient: _______________________95% CI: ________________________ p-value: _______
Findings: ____
Limitations: ____

Inclusion Criteria

Patients on methadone maintenance treatment

RCT or observational study design

Cannabis measured as a predictor variable

Exclusion Criteria

Patients on any other opioid substitution therapy

Methadone being used for purposes other than maintenance treatment (i.e. recreational or detoxification)

Cannabis measured as an outcome variable

Study does not measure a primary (illicit opioid use, treatment retention) or secondary (criminal activity, jail time, polydrug use, and injecting behaviours) outcome variables

Additional Comments:

____

____

____

____

____
